# Malignant ureteral obstruction: experience and comparative analysis of metallic versus ordinary polymer ureteral stents

**DOI:** 10.1186/s12957-019-1608-6

**Published:** 2019-04-30

**Authors:** Yue Chen, Cheng-yi Liu, Zhi-hong Zhang, Peng-cheng Xu, De-gang Chen, Xin-huan Fan, Ji-ci Ma, Yi-peng Xu

**Affiliations:** 1 Department of Urology, The Second Hospital of Tianjin Medical University, No.23, Pingjiang Road, Hexi Disctrict, Tianjin, 300211 China; 2 Department of Urology, Lu’an Affiliated Hospital of Anhui Medical University, Lu’an, 237000 Anhui China; 3 Institute of Urology, Zhejiang Cancer Hospital, Hangzhou, 310000 ZheJiang China

**Keywords:** Metallic stent, Contrast study, Malignant, Ureteral obstruction

## Abstract

**Background:**

To study the outcome and experience of using metallic stents in treating patients with malignant ureteral obstruction (MUO).

**Methods:**

Seventy-six patients with MUO were assigned to the metallic stent group (MSG) or the ordinary polymer stent group (OPSG) according to the different materials. The success rate of the operation, duration of operation, patency rate serum creatinine values ,postoperative complications and QOL scores were compared between the two groups.

**Results:**

In the OPSG and MSG, the success rates of the operation were 95.5% and 96.9%, respectively, and the durations of the operation were 20.6 ± 2.2 min and 50.9 ± 10.3 min (*P* < 0.01), respectively. There was no significant difference between the groups in serum creatinine values at 3 days after the operation (*P* > 0.05); however, the creatinine values at 3 days after the operation decreased significantly compared with those before the operation (*P* < 0.01). In the OPSG, there was no significant difference in creatinine values between 3 days and 6 months after operation, while the creatinine values 1 year after operation were increased significantly compared to those at 3 days after the operation (*P* < 0.05). In the MSG, there was no significant difference among creatinine values at different intervals (*P* > 0.05). The total rate of post-procedural complication was lower in the MSG than that in the OPSG(*P* < 0.05). There was no significant difference in the QOL score between the two groups before the operation (*P* > 0.05); however, the QOL scores at 6 months and 1 year after the operation were higher in the MSG than that in the OPSG(*P* < 0.05). In the MSG, there was no significant difference in the QOL score between preoperation and 6 months after surgery. Similarly, there was also no difference in the QOL score between 6 months after surgery and 1 year after surgery(*P* > 0.05). On the contrary, the differences of QOL score in the OPSG group were much significant between disparate time intervals (*P* < 0.05).

**Conclusions:**

For patients with MUO who require long-term retention of the stent, metallic stents with longer indwelling time are superior to ordinary polymeric stents.

## Introduction

Ureteral obstructions secondary to malignant tumour increase each year, mainly due to the following factors. First, primary tumours infiltrate the ureteral wall; second, tumours or metastatic lesions oppress the ureter; third, the swollen lymphonodus wraps the ureter [1]; fourth, ureter distortion and lumen stenosis occurs due to oedema and retroperitoneal fibrosis after radiotherapy; and fifth, the elasticity of the ureter is weakened after radiotherapy, thereby affecting its peristaltic urine delivery function [2]. One or both sides could be involved. Clinical removal of the obstruction and rapid improvement of renal function are the primary principles for treatment. In this context, ordinary ureteral stents have poor therapeutic effects and require replacement within a short time, which could lead to secondary trauma; thus, it is necessary to choose methods that maximize the drainage patency and quality of life for the patient, in order to minimize discomfort during stent placement and replacement [3]. Metallic ureteral stent can make up for the above deficiency. In this study, we compared the advantages of new metallic Resonance® stents (Cook Medical, Bloomington, IN, USA) with that of ordinary polymer stents according to large amounts of data, such as serum creatinine values, postoperative patency, postoperative complications, QOL scores, and the beneficial time from surgery. In addition, we obtained the objective evaluation of patients on the metallic stent with QOL score and summarized the surgical experience of catheterization.

## Material and methods

### Patient characteristics

Data were retrospectively obtained from the medical records of patients with MUO who were treated during the period of June 2014 to August 2018 at the Second Hospital of Tianjin Medical University, Lu'an Affiliated Hospital of Anhui Medical University, and Zhejiang Cancer Hospital. This retrospective study was approved by the institutional review board in our hospital. Patients were informed of the risks and benefits of participation in the study; each provided written, informed consent before enrolment. Based on the different ureteral stent materials, 76 patients were divided into the metallic stent group (MSG) and the ordinary polymer stent group (OPSG), as shown in Table 1.

**Table 1:** Patient characteristics of the two groups

Categories	MSG (*n* = 32)	OPSG (*n* = 44)	*P* value
Gender			
Male	13	18	0.980
Female	19	26	
Age (years)	66 (43~81)	61 (49~78)	0.722
SOPT			
Pelvis			
Cervical cancer	10	14	0.281
Ovarian cancer	6	9	
Rectal cancer	7	5	
Bladder cancer	3	6	
Prostate cancer	2	2	
PMT	1	0	
Abdomen			
Gastric cancer	1	7	
MRT	2	1	
SOUO			
Unilateral	22	29	0.795
Bilateral	10	15	
PC (μmol/L)			
Normal	3	1	0.171
Abnormal	29	43	
Methods			
Ant			
PN	9	0	< 0.001
Ret			
Ureteroscope	21	33	
Cystoscope	1	9	
Failure	1	2	
Anaesthesia			
GA	5	8	0.770
NO-GA	27	36	

In the MSG, which comprised 32 patients, 3 different surgical methods were adopted. One case involved catheterization failure, defined as follows: First, all patients included initially accepted retrograde ureteroscope or cystoscope catheterization. Those who could not receive catheterization accepted the percutaneous nephroscope anterograde catheterization; if that failed, the case was recorded as failed catheterization, and nephrostomy was conducted. Second, retrograde ureteroscope or cystoscope catheterization was successful, but the stent position was not good after re-examination; moreover, renal functional improvement was not ideal. In the OPSG, which comprised 44 patients, there were 2 cases of failed catheterization, followed by nephrostomy. The evaluation criteria for inadequate stent drainage after surgery were as follows: with the exclusion of hypertensive nephropathy, diabetic nephropathy, and other kidney damage factors, the patient’s serum creatinine values increased and hydronephrosis became aggravated, as shown by the urinary colour ultrasound or other imaging examinations. For such patients, the stent should be changed before the expected time; if this fails, a nephrostomy was performed.

### Operation methods

For OPSG, ureteroscope and cystoscope catheterization were performed in a manner similar to that of traditional surgical methods. For MSG, the following methods were used:

1. Ureteroscopy catheterization: In the lithotomy position, the guide wire was passed through the ureter lumen into the kidney pelvis with a ureteroscope, followed by a coaxial introduction of the Resonance® sheath and catheter. The guidewire and catheter were then exchanged for the closed-ended metallic stent through the sheath, with the aid of the catheter as a pusher. The sheath was then finally removed to leave the stent in position.
2. Percutaneous nephroscope anterograde catheterization: In the prone position, guided by ultrasound, the target calyx was punctured. After establishing percutaneous nephroscope channels, the guide wire was anterogradely placed into the bladder with a ureteroscope. Subsequently, the outer sheath along the guide wire was placed into the bladder, and the metallic stent was then placed along the sheath into the bladder and formed a circle in the kidney pelvis.
3. Cystoscope ureteral catheterization: In the lithotomy position, local anaesthesia was conducted. The method of catheterization was similar to that of the ureteroscope approach.

### Statistical analysis

SPSS 16.0 software (IBM Corp, Armonk, NY, USA) was used to analyse the data. The data were expressed as $\overline{x}\pm s$. The Mann-Whitney test, chi-square test, and Cox regression analysis were used. *P* < 0.05 was considered statistically significant.

## Results

### Comparison of success rate and duration of operation between the two groups

**Fig. 1:**
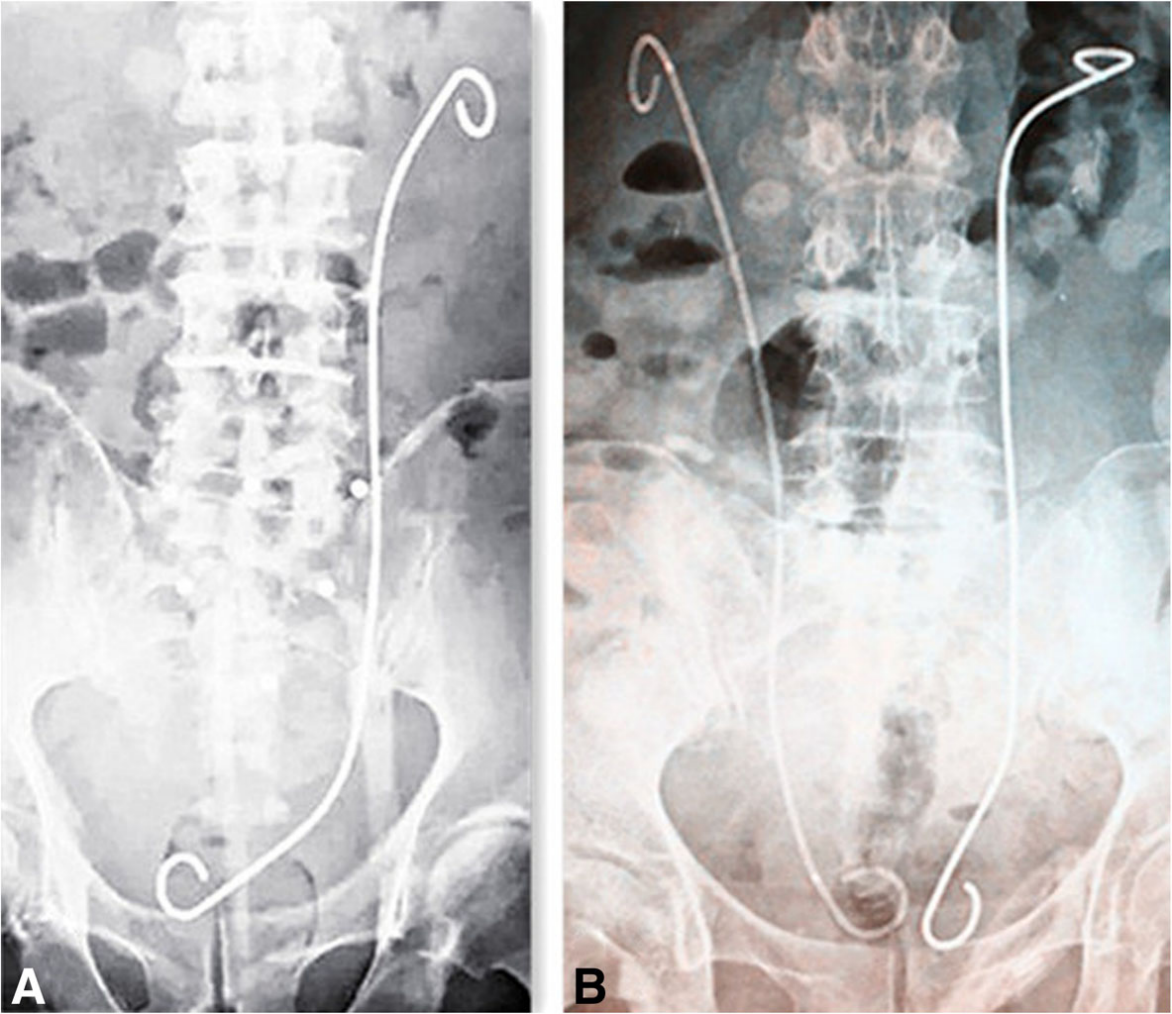
Metallic stent on the left (**a**) and metallic stent on the left with ordinary polymeric stent on the right (**b**)

**Fig. 2:**
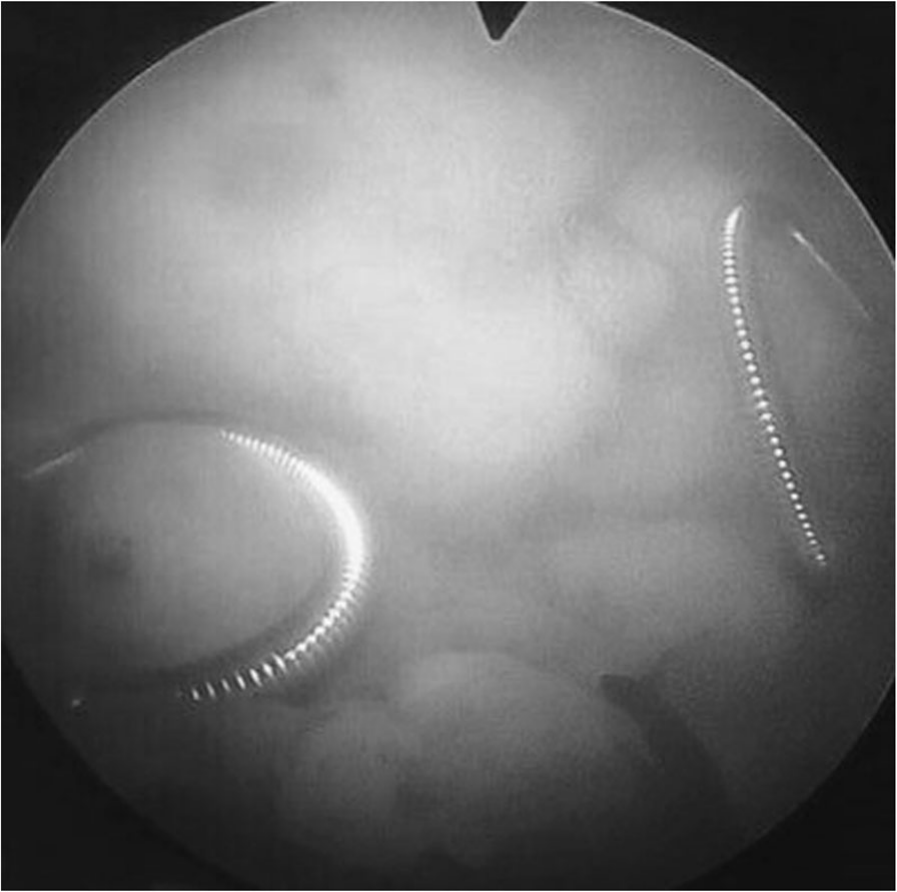
Metallic stents can be seen in the bladder on both sides with ureteroscope

The success rate and duration of operation for each group are shown in Table 2. For the OPSG, which comprised of 44 patients, a total of 56 stents were indwelled in the first catheterization, and there were 2 failure cases. Regarding the replacement of the stents within the next 1 year, there were 5 failed cases. For the MSG, which comprised of 32 patients, a total of 41 stents were indwelled; there are 31 successful cases (Figs. 1 and 2) as well as 1 failed case. Considering the economic aspects for patients with bilateral hydronephrosis, there were three cases in the MSG where the metallic stent was accepted on the side with better kidney function, whereas ordinary polymeric stent was accepted on the other side. The duration of operation for the MSG was longer than that for the OPSG (*P* < 0.01), according to statistical analysis.

**Table 2:** Success rate and duration of operation of the two groups

Group	Cases	Successful cases	Success rate	Duration of operation (min)	*P* value
MSG	32	31	96.9% (31/32)	50.9 ± 10.3	
OPSG	44	42	95.5% (42/44)	20.6 ± 2.2	< 0.01

Duration of operation is shown only for cases with successful surgery

### Comparison of serum creatinine values before and after operation between the two groups

Serum creatinine values before the operation and 3 days, 6 months, and 1 year after the operation are shown in Table 3. The number of cases was modified due to death, failure of normal follow-up and catheterization, and a lack of sufficient time for statistical analysis, as shown in Table 4. Hydronephrosis of these postoperative patients was improved, as shown in the imaging manifestations; lumbago and pyrexia were reduced significantly or disappeared. There was no significant difference in serum creatinine values between the two groups at 3 days after the operation (*P* > 0.05); however, the creatinine values at 3 days after the operation decreased significantly compared with those before the operation (*P* < 0.01). In the OPSG, there was no significant difference in creatinine values between 3 days and 6 months after surgery, while creatinine values at 1 year after surgery were increased compared with those at 3 days after surgery (*P* < 0.05). In the MSG, there were no significant differences among creatinine values at 3 days, 6 months, and 1 year after the operation (*P* > 0.05). The average duration of follow-up was 12 months, during which ordinary polymer stents were generally replaced within 1–3 months and metallic stents were replaced within approximately 12 months; the longest retention time was approximately 2 years. In the MSG, outpatient re-examinations showed no fracture, translocation, or dysfunction in the metallic stents. Among all patients who were followed-up, six experienced intermittent haematuria, four experienced repeated urinary tract infections after surgery, and one exhibited stones attached to metallic stents.

**Table 3:** Results of serum creatinine (μmol/L) detection of the two groups ($\overline{x}\pm s$)

	Creatinine before operation	3 days after	6 months after	1 year after
MSG	393.9 ± 93.9 (*n* = 31)	121.0 ± 19.8 (*n* = 31)	121.3 ± 22.2 (*n* = 27)	18.2 ± 22.2 (*n* = 23)
OPSG	377.5 ± 38.4 (*n* = 42)	119.3 ± 14.5 (*n* = 42)	159.0 ± 34.9 (*n* = 36)	235.9 ± 39.0 (*n* = 28)

Serum creatinine values are shown only for cases with successful surgery

**Table 4:** Reasons for altered numbers of cases at different stages

Group		3 days after	6 months after	1 year after
MSG	Death	0	1	4
	Failure of normal follow-up	0	1	0
	Failure of catheterization	1	/	/
	Failure of statistical time	0	2	/
	Statistical cases	31	27	23
OPSG	Death	0	2	3
	Failure of normal follow-up	0	3	1
	Failure of catheterization	2	1	1
	Statistical cases	42	36	28

### Comparison of patency rate 1 year after operation between the two groups

**Fig. 3:**
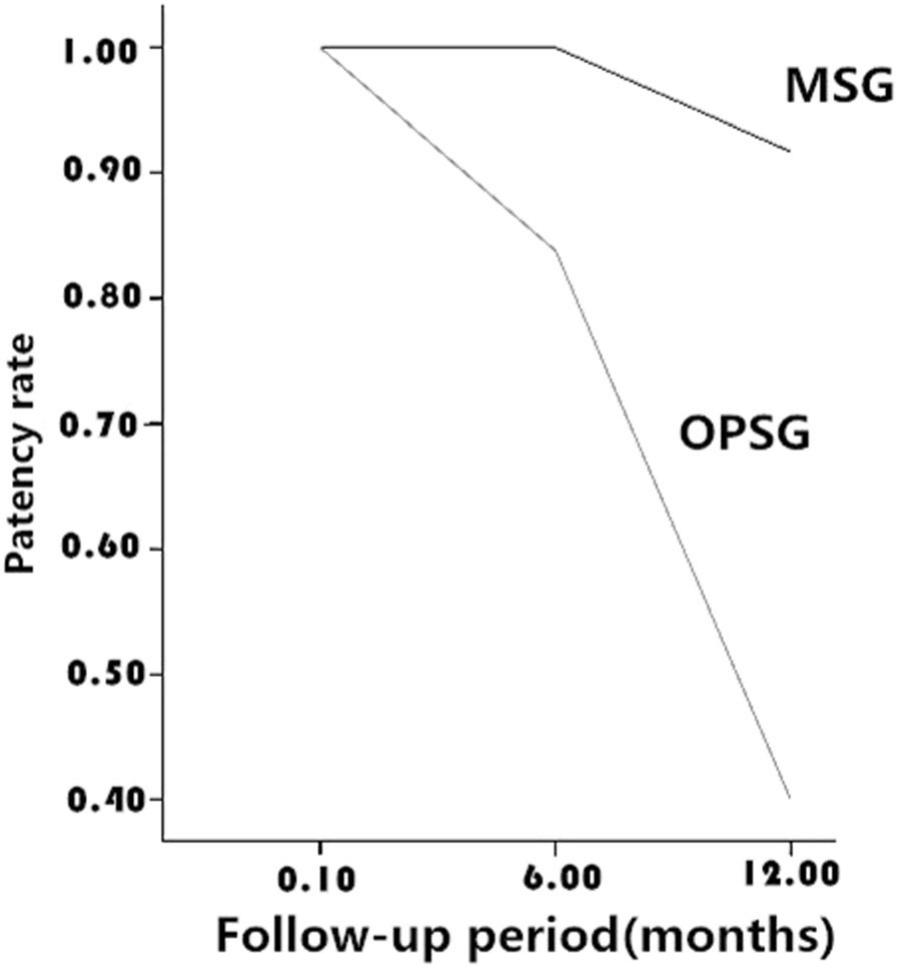
Comparison of the patency rate between the two groups, 1 year after operation

In the OPSG, the patency rates at 3 days, 6 months, and 1 year after the operation were 100% (42/42), 83.8% (31/37), and 40.0% (12/30), respectively (Fig. 3). There were a total of five deaths; three of these (one within 6 months and two within 1 year after surgery) exhibited renal impairment before death. In the MSG, the patency rates at 3 days, 6 months, and 1 year after operation were 100% (31/31), 100% (28/28), and 91.7% (22/24), respectively (Fig. 3). Two of these patients exhibited renal impairment within 1 year after the operation, with increased creatinine values and aggravated hydronephrosis as shown by urinary colour ultrasound. One of the cases was considered to be the result of the obstruction of urinary blood clots, since the KUB showed that the metallic stents were in good locations with normal shapes. Therefore, after symptomatic treatment, the renal function gradually improved during follow-up. The other case involved death due to lumen blocking caused by bladder cancer invading the ureter. Four other deaths involved no obvious damage to renal function.

### Comparison of post-procedural complication, QOL score, and survival risk between the two groups

The post-procedural complication and QOL score of different stages for each group are shown in Tables 5, 6, and 7. The total rate of post-procedural complication was lower in the MSG than that in the OPSG (*P* < 0.05). There was no significant difference in QOL score between the two groups before the operation (*P* > 0.05); however, the QOL scores at 6 months and 1 year after the operation were higher in the MSG than that in the OPSG (*P* < 0.05). In the MSG, there was no significant difference in the QOL score between preoperation and 6 months after surgery. Similarly, there was also no difference in the QOL score between 6 months after surgery and 1 year after surgery (*P* > 0.05). On the contrary, the differences of QOL score in the OPSG group were much significant between disparate time intervals (*P* < 0.05).

**Table 5:** Results of post-procedural complications of the two groups

Group	ISOB	UTI	Lumbago	APOS	Haematuresis	COS	total	*P* value
MSG (*n* = 30)	2	2	1	0	6	0	11	
OPSG (*n* = 33)	3	5	1	3	5	4	21	0.032

Post-procedural complications are shown only for cases with normal follow-up and successful surgery

**Table 6:** Results of QOL score of the two groups ($\overline{x}\pm s$)

QOL Score						
Group	Before operation	6 months after	*P* value	6 months after	1 year after	*P* value
MSG	29.1 ± 2.5	30.9 ± 2.8	0.325	30.9 ± 2.8	30.7 ± 3.1	0.845
OPSG	28.4 ± 1.5	23.6 ± 1.8	0.002	23.6 ± 1.8	21.3 ± 1.1	0.01

**Table 7:** Comparison of QOL score between the two groups

QOL score			
Time	MSG	OPSG	*P* value
Before operation	29.1 ± 2.5	28.4 ± 1.5	0.167
6 months after	30.9 ± 2.8	23.6 ± 1.8	< 0.001
1 year after	30.7 ± 3.1	21.3 ± 1.1	< 0.001

**Fig. 4:**
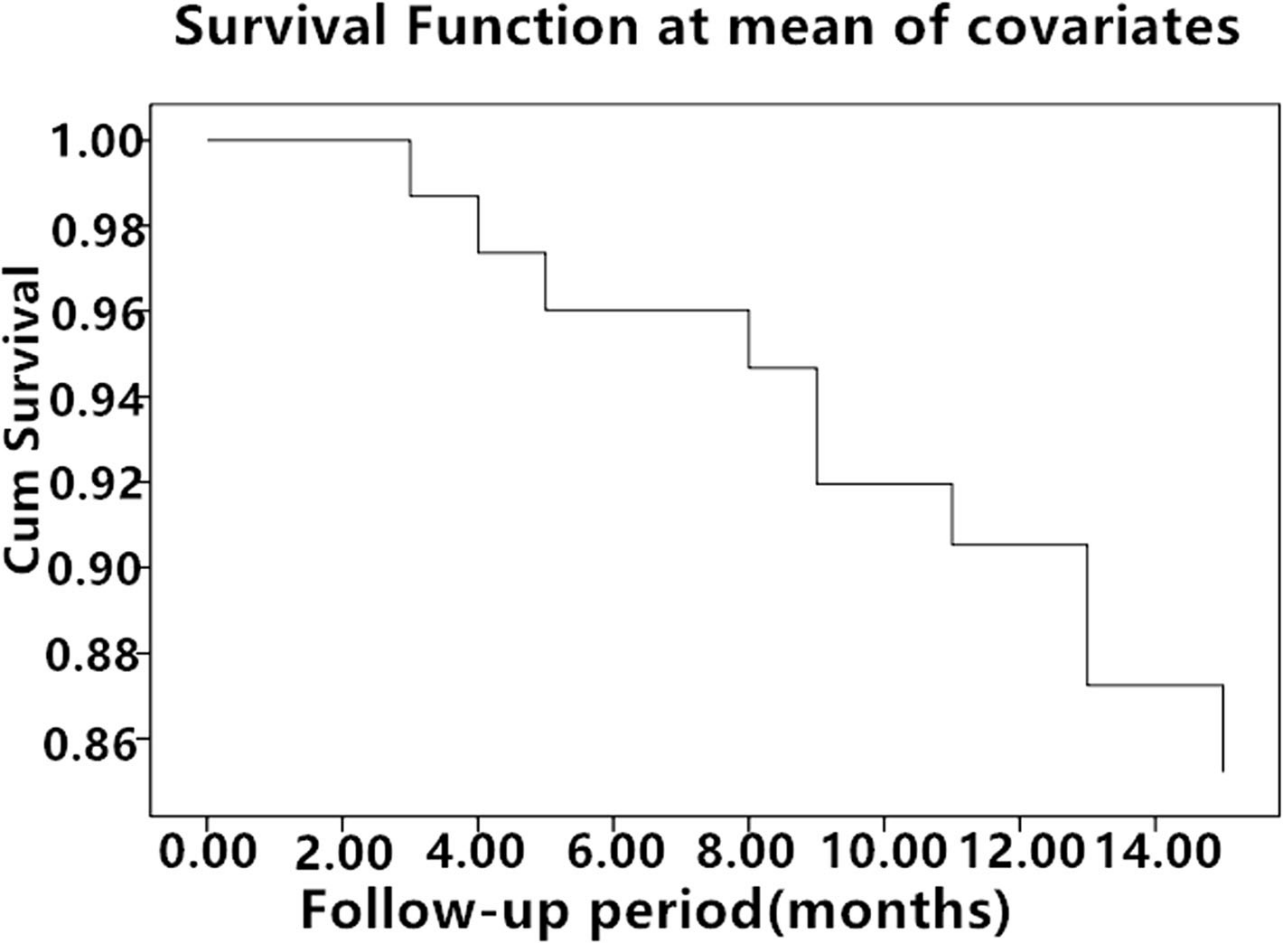
Survival rates decreased with prolonged follow-up

The Cox multivariate analysis of potential survival risk did not yield any endpoints of statistical significance including gender, age at diagnosis, tumour location, site of ureteral obstruction, preoperative creatinine, stent type, and anaesthesia in spite of reduced survival rates with prolonged follow-up (Table 8 and Fig. 4).

**Table 8:** Cox multivariate analysis of survival risk of patients with MUO

Variable	*P* value
Gender	0.283
Age	0.064
SOPT (abdomen, pelvis)	0.870
SOUO (bilateral, unilateral)	0.159
PC	0.399
Stent type	0.753
Anaesthesia	0.250

## Discussion

Malignant tumours causing ureteral obstruction are mainly from the abdomen, pelvis, and retroperitoneum; these include rectal cancer, colon cancer, gastric cancer, cervical cancer, ovarian cancer, bladder cancer, and prostate cancer. Methods for removing malignant external compressive ureteral obstructions include ureteral stenting and nephrostomy. Since nephrostomy causes inconvenience and trauma, ureteral stenting is often used. Three technical parameters are critical for ideal ureteral stent performance: design, materials, and surface coating [4], and thus, it can be divided into ordinary polymer stent, strengthened polymer stent, metallic stent, coated stent, and drug-eluting stent. Ordinary ureteral stents are made with a mixture of polyethylene and polyurethane [5]; thus, they are limited by their own composition, including poor compression resistance. When used for the treatment of malignant external compressive ureteral obstructions, such stents easily cause inadequate drainage, as well as a short replacement cycle. Therefore, it is difficult to ensure long-term and efficient drainage, and new ideal ureteral stents are needed. The compression resistance of strengthened polymer stent is better than that of ordinary polymer stent; however, it may still lead to poor drainage under higher pressure. The metallic stent may be finally selected as a last resort and has been reported to be safe and effective in the treatment of MUO [6–13]. Christman et al. evaluated the resistance to radial compression of various stents, Silhouette, Sof-Curl, Tecoflex, Resonance, Polaris Ultra, and Percuflex. The results showed that greater force was needed to compress the Resonance and Silhouette stents compared to others [14].

In this study, we used the Resonance® metallic ureteral stent, which is a hollow, spiral-shaped, full-length metal stent with the same shape as the double J catheter. It has the following advantages: first, it is made of nickel-cobalt-chromium-molybdenum alloy, which has high biocompatibility and less capsid, such that it is not prejudiced against MRI examination; second, its dense, hollow, spiral structure can inhibit endogenous tissue growth and can provide double drainage from the spiral outer surface and the lumen; third, its radial compressive resistance is > 68 kg, which is much higher than that of traditional stents (4~11 kg) and other metal stents [15]; fourth, it is soft, with a certain elasticity and less abnormal sensation; fifth, its indwelling time is more than 12 months, thus reducing the pain caused by stent replacement; and sixth, the stent and its accessories can clearly be shown through X-ray and B-ultrasound, facilitating monitoring and re-examination. Considering the high-pressure resistance, good drainage effect, longer replacement period, and advantages of ensuring the patient quality of life, the Resonance® metallic ureteral stent has become an ideal stent for removing MUO; for patients with shorter life expectancy, it can even be a permanent stent for treatment.

The patency rate of metallic stents for the treatment of MUO lies between 37% and 100% [16–21], and the success rate of the operation is 96% [22], according to literature reports. In our study, the success rate of metallic stent catheterization was 96.9%. In the two groups, hydronephrosis improved, as shown by imaging; moreover, there was no significant difference in serum creatinine values between the groups (*P* > 0.05), while creatinine values at 3 days after the operation in each group were significantly lower than those before the operation (*P* < 0.01). This finding indicated that catheterization of either metallic stents or ordinary polymeric stents could enable effective hydronephrosis drainage and improve short-term renal function. As the statistical analysis shows, there was no significant difference in creatinine values among 3 days, 6 months, and 1 year after operation in the MSG (*P* > 0.05). Furthermore, in the OPSG, there was no significant difference in creatinine values between 3 days and 6 months after operation (*P* > 0.05); however, the creatinine values at 1 year after operation were significantly higher than those at 3 days after operation (*P* < 0.05). The patency rate decreased to 40.0%, which was in striking contrast to the 91.7% observed in the MSG; this finding indicated that over 6 months, both the metallic stent and ordinary polymeric stent maintain a high drainage patency rate. However, 1 year later, even ordinary polymeric stents with regular replacement could not assure a higher patency rate, and the replacement success rate was gradually decreased. The advantages of metallic stents are notable. Although the duration of the operation of metallic stent catheterization was longer than that of the ordinary polymeric stent (*P* < 0.01), this difference may be related to the surgeon’s proficiency. Overall, therefore, metallic stents are preferred for patients with malignant external compressing ureteral obstructions.

**Fig. 5:**
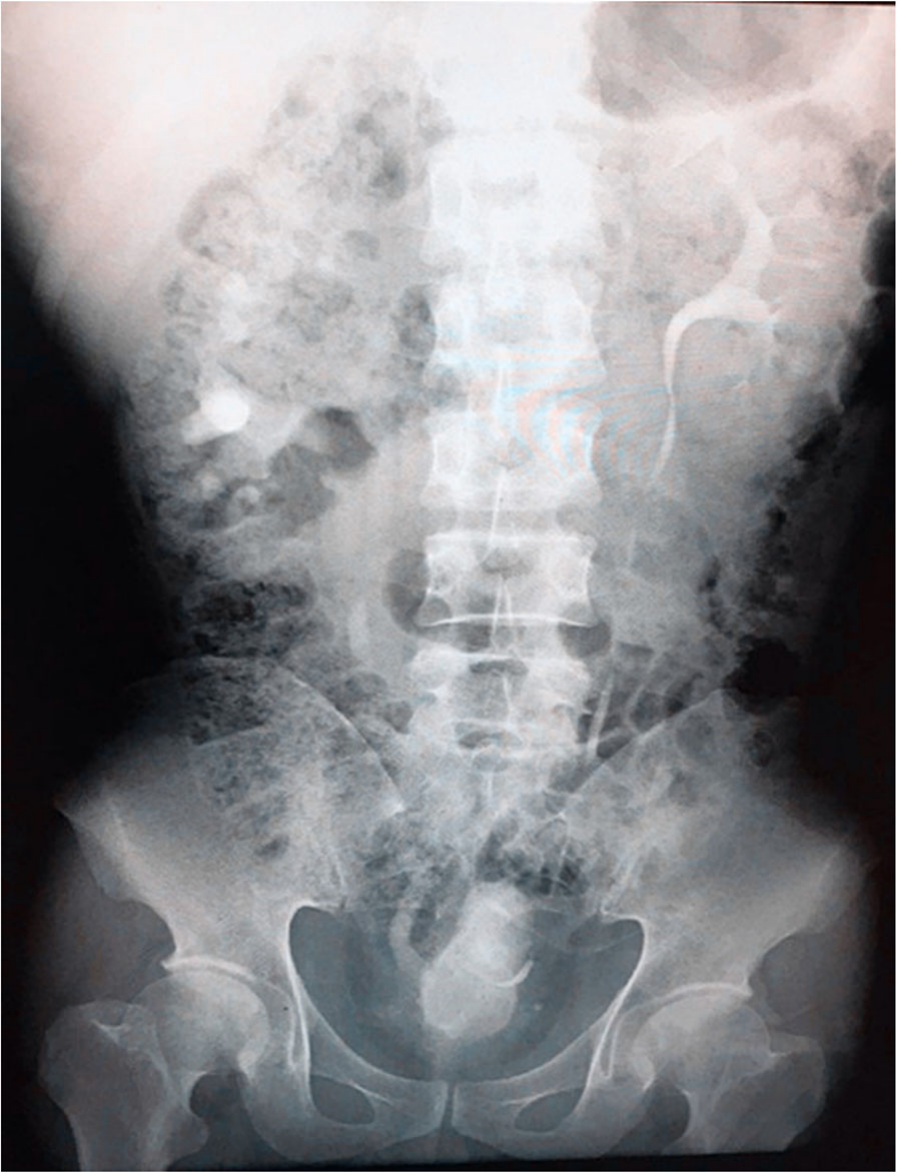
Altered bladder shape after radical resection of rectal carcinoma, shown by urography

**Fig. 6:**
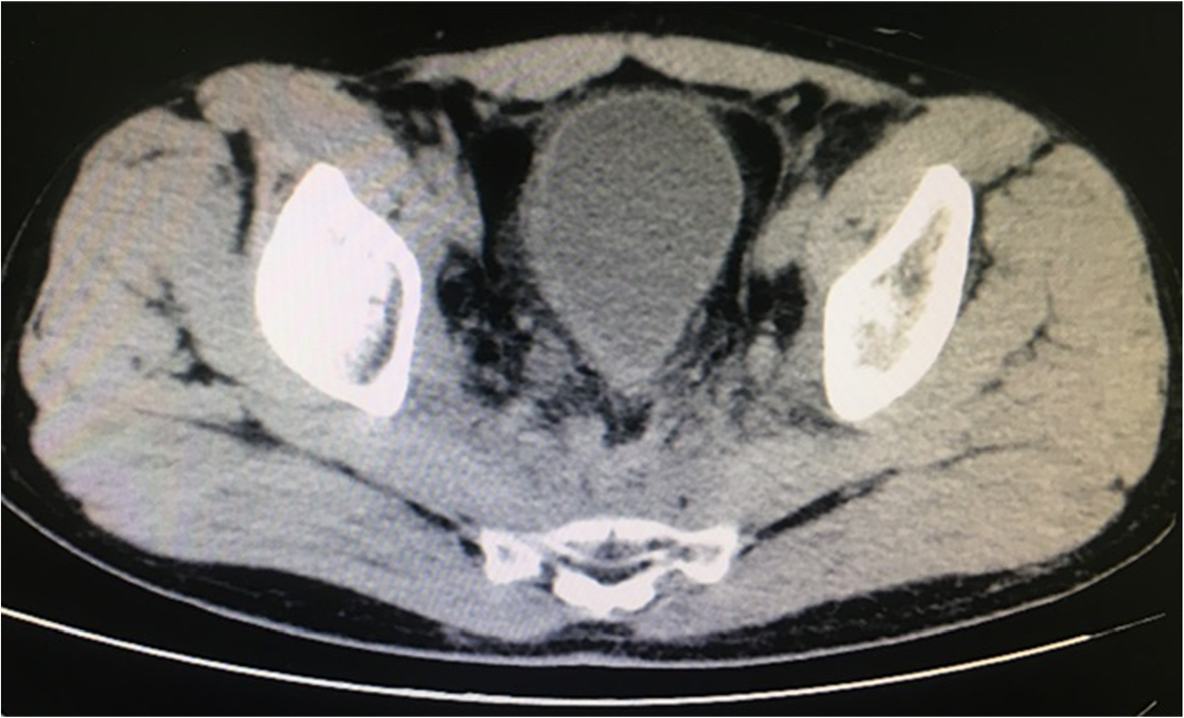
Altered bladder shape after radical resection of rectal carcinoma, shown by computed tomography

**Fig. 7:**
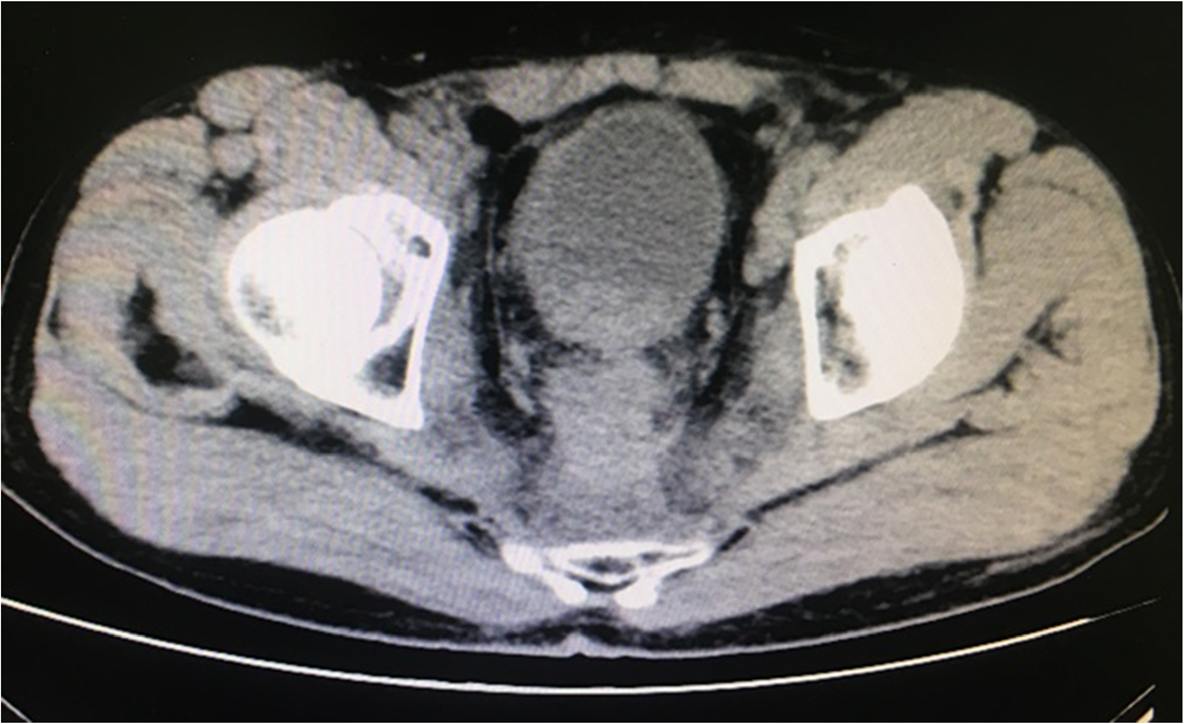
Tumour invading the bladder neck and the right wall

Thus far, reports of surgical experience sharing regarding metallic stent catheterization are not too much. To our knowledge, the success rate of catheterization has no relation to the type of stent but is related to the patient’s objective condition. The main reasons for failed catheterization are as follows: first, bladder shape is altered and the ureteral orifice moves with pelvic tumour compression, or after uterine and rectal tumour surgery, thereby causing an undiscoverable ureteral orifice (Figs. 5 and 6); second, the tumour infiltrates the ureteral orifice, which leads to an undiscoverable ureteral orifice or inability of the guide wire to enter the ureteral lumen (Fig. 7); third, patients exhibit vesicovaginal fistula, in which the bladder does not fill; and fourth, ureteral lumen distortion or metastatic lesions (e.g. lymphonodus) can exhibit pressure on the ureteral lumen. Our experiences suggest the following approach: initially, each patient should accept CTU, magnetic resonance hydrography, or urinary contrast, in order to assess ureteral status before surgery. In addition, stenotic segments often arise from the lower ureter. In ureterostenosis, an extension can be made during the operation with a ureteral catheter or renal sheath dilator, followed by catheterization. This often leads to success. With a ureteroscope horizontally rotated and extruded with a specific vertical strength, the stenotic segments could also be broken through and the ureteral injury or fracture could generally be avoided. Finally, when the catheterization fails for the reasons above, there are various flexible methods for management, such as percutaneous nephroscope anterograde catheterization for situations involving an undiscoverable ureteral orifice, or a preset micro-nephrostomy tube (F6 or F7) with methylene blue injection during the operation to aid in locating the ureteral orifice via ureteroscope. In the clinic, we usually choose ureteroscope or cystoscope catheterization, which may cause relatively small trauma; most of these surgeries are successful. In addition, the method of percutaneous nephroscope anterograde catheterization has been conducted entirely due to undiscoverable ureteral orifice or failure to enter the ureter via ureteroscope.

Those who have internal metallic stents need regular follow-up and should replace the stents within 1 year to avoid stone formation. Some patients who have rapid tumour progression or infiltrated ureteral lumen, or who are susceptible to stones should replace the stents within 6 months [17]. During our follow-up, the average indwelling time of the ordinary polymer stent was only 3 months, while the indwelling time of the metallic stent was approximately 12 months, and even nearly 2 years. As the statistical analysis has shown, the total rate of post-procedural complication was lower in the MSG than that in the OPSG(*P* < 0.05). The outpatient re-examinations showed no fracture, translocation, or dysfunction in the metallic stents. There was no significant difference in the QOL score between the two groups before the operation(*P* > 0.05). However, the QOL scores at 6 months and 1 year after the operation were higher in the MSG than that in the OPSG (*P* < 0.05). In the MSG, there was no significant difference in the QOL score between preoperation and 6 months after surgery. Similarly, there was also no difference in the QOL score between 6 months after surgery and 1 year after surgery(*P* > 0.05). On the contrary, the differences of QOL score in the OPSG group were much significant between disparate time intervals (*P* < 0.05). We think there were several reasons for the lower QOL score of most patients with ordinary polymer stents. Firstly, because the QOL score was greatly influenced by the progress of primary tumour and individual psychological factors, frequent replacement of the stent might result in worry and fear. Secondly, nephrostomy leads to a low quality of life for those patients with failed catheterization due to inconvenience and trauma. Thirdly, family conflicts may occur due to repeated hospitalizations. Lastly, postoperative complications were the important factors. The Cox multivariate analysis of potential survival risk did not yield any endpoints of statistical significance including gender, age at diagnosis, tumour location, site of ureteral obstruction, preoperative creatinine, stent type, and anaesthesia in spite of reduced survival rates with prolonged follow-up (Fig. 4). Additionally, we further analyzed the reasons why stent type did not affect the survival of patients. We do not deny the fact that metallic stent can indeed reduce post-procedural complication, prolong the period of stent replacement, and improve the quality of life. Nevertheless, nephrostomy can still maintain good renal function irrespective of the inferior quality of life for those patients with ordinary polymer stent and even failed catheterization in the replacement process. What really affected survival was the degree for the progression of primary tumour. The metallic ureteral stent has limitations; for example, it has high costs for a single operation. However, some studies have shown that the patients experience reduced financial burden because of the reduced frequency of replacement, despite the high single-operation costs [23–26]. Given the cost-effectiveness of metallic stents, they are more suitable for long-term treatment.

## Conclusions

We conclude that the Resonance® metallic stent is safe and effective for treating MUO. For patients who require long-term stent retention, metallic ureteral stents with good drainage effect, longer indwelling time, and lower cost are superior to ordinary polymeric stents.

## Abbreviations

Ant: Anterograde
APOS: Abnormal position of stent
COS: Capsid of stent
GA: General anaesthesia
ISOB: Irritation sign of bladder
MRT: Malignant retroperitoneal tumour
MSG: Metallic stent group
MUO: Malignant ureteral obstruction
OPSG: Ordinary polymer stent group
PC: Preoperative creatinine
PMT: Pelvic malignancy tumour
PN: Percutaneous nephroscope
Ret: Retrograde
SOPT: Site of primary tumour
SOUO: Site of ureteral obstruction
UTI: Urinary tract infection
